# Molecular Evidence for Natural Hybridization between *Cotoneaster dielsianus* and *C. glaucophyllus*

**DOI:** 10.3389/fpls.2017.00704

**Published:** 2017-05-09

**Authors:** Mingwan Li, Sufang Chen, Renchao Zhou, Qiang Fan, Feifei Li, Wenbo Liao

**Affiliations:** ^1^Guangdong Key Laboratory of Plant Resources, Key Laboratory of Biodiversity Dynamics, Conservation of Guangdong Higher Education Institutes, Sun Yat-sen UniversityGuangzhou, China; ^2^State Key Laboratory of Environmental Criteria and Risk Assessment, Chinese Research Academy of Environmental SciencesBeijing, China; ^3^College of Life and Environmental Sciences, Minzu University of ChinaBeijing, China

**Keywords:** *Cotoneaster*, natural hybridization, low-copy nuclear genes, chloroplast DNA, polyploidy, apomixes

## Abstract

Hybridization accompanied by polyploidization and apomixis has been demonstrated as a driving force in the evolution and speciation of many plants. A good example to study the evolutionary process of hybridization associated with polyploidy and apomixis is the genus *Cotoneaster* (Rosaceae), which includes approximately 150 species, most of which are polyploid apomicts. In this study, we investigated all *Cotoneaster* taxa distributed in a small region of Malipo, Yunnan, China. Based on the morphological characteristics, four *Cotoneaster* taxa were identified and sampled: *C. dielsianus, C. glaucophyllus, C. franchetii*, and a putative hybrid. Flow cytometry analyses showed that *C. glaucophyllus* was diploid, while the other three taxa were tetraploid. A total of five low-copy nuclear genes and six chloroplast regions were sequenced to validate the status of the putative hybrid. Sequence analyses showed that *C. dielsianus* and *C. glaucophyllus* are distantly related and they could be well separated using totally 50 fixed nucleotide substitutions and four fixed indels at the 11 investigated genes. All individuals of the putative hybrid harbored identical sequences: they showed chromatogram additivity for all fixed differences between *C. dielsianus* and *C. glaucophyllus* at the five nuclear genes, and were identical with *C. glaucophyllus* at the six chloroplast regions. Haplotype analysis revealed that *C. dielsianus* possessed nine haplotypes for the 11 genes, while *C. glaucophyllus* had ten, and there were no shared haplotypes between the two species. The putative hybrid harbored two haplotypes for each nuclear gene: one shared with *C. dielsianus* and the other with *C. glaucophyllus*. They possessed the same chloroplast haplotype with *C. glaucophyllus*. Our study provided convincing evidence for natural hybridization between *C. dielsianus* and *C. glaucophyllus*, and revealed that all hybrid individuals were derivatives of one initial F1 via apomixes. *C. glaucophyllus* served as the maternal parent at the initial hybridization event. We proposed that anthropological disturbance provided an opportunity for hybridization between *C. dielsianus* and *C. glaucophyllus*, and a tetraploid F1 successfully bred many identical progenies via apomixis. Under this situation, species integrity could be maintained for these *Cotoneaster* species, but attentions should be kept for this new-born hybrid.

## Introduction

Hybridization, previously viewed as a mere side branch or noise of evolution, is now recognized as a major evolutionary force and a significant portion of speciation (e.g., Arnold, 1997; Rieseberg and Willis, 2007; Soltis and Soltis, 2009; Soltis et al., 2014). The process of hybridization can help us understand the origin of adaptations, the maintenance of plant diversity, and the formation of new species. As early as 1917, Winge first introduced a theory linking the formation of hybridization and the development of polyploids, proposing that reproductive isolation could be rapidly established between new polyploids and their parental species, so that new polyploid hybrid species could arise in just a few generations (Winge, 1917). Several plant species have originated via hybridization and polyploidy within the past 150 years, such as *Spartina anglica* (Ainouche et al., 2003), *Senecio cambrensis* and *S. eboracensis* (Abbott and Lowe, 2004), *Cardamine schultzii* (Urbanska et al., 1997), and *Tragopogon mirus* and *T. miscellus* (Soltis et al., 2004). It was also proposed that “allopolyploidy, perhaps more than any other process, has played a major role in the origin of many species and thus has driven and shaped the evolution of vascular plants” (Feldman and Levy, 2005) and many angiosperms are ultimately of ancient polyploid origin (Wagner and Wagner, 1980).

The production of viable progenies is a key for the establishment of a hybrid lineage. Allopolyploids may frequently produce pollen with meiotic irregularities, leading to partial or complete sterility of the progenies (Comai et al., 2003; Comai, 2005). A potential evolutionary solution to this problem is asexual reproduction, i.e., apomixis or agamospermy (Asker and Jerling, 1992; Sochor et al., 2015). With usually uniparental reproduction, lowered cost of sex, maintenance of adapted genotypes and occasional seed reproduction (Hörandl, 2006), many apomictic plants can achieve great ecological and evolutionary success. Apomixis has been well documented in numerous genera of Rosaceae, particularly *Cotoneaster* (Nybom and Bartish, 2007), *Crataegus* (Lo et al., 2009), *Rubus* (Sochor et al., 2015), *Sorbus* (Robertson et al., 2010; Ludwig et al., 2013), and *Potentilla* sensu lato (Morgan et al., 1994).

Derived from hybridization and chromosome doubling, allopolyploids always display intermediate morphologies compared to their parents. Many allopolyploid apomicts are facultative, and their backcrossing with sexual relatives is hypothesized to lead to multiple evolutionary origins for apomictic lineages; the morphological differences between these species with apomixis can be very small (Van der Hulst et al., 2000; Paun et al., 2006; Sochor et al., 2015). The interplay of hybridization, polyploidy and apomixis generated a great number of described species in Rosaceae, whose taxonomic classification has been a challenging task for generations of researchers. These species are not easily distinguishable and have only relatively minor morphological differences.

As a typical example, the genus *Cotoneaster* Medik. (Rosaceae, subtribe Malinae) is fraught with hybridization accompanied by polyploidy and apomixis. The genus occurs throughout Europe, North Africa and temperate areas of Asia excluding Japan. The Himalayas and neighboring mountains in Yunnan and Sichuan of China are the most important species diversity center for this genus. Furthermore, the majority (70%) of *Cotoneaster* taxa have so far proven to be tetraploid (2*n* = 68), which are mostly in-breeding apomictic taxa; only 10% are diploid (2*n* = 34) (Fryer and Hylmö, 2009). Observations from seedling morphology and embryo sac development also revealed that apomictic breeding systems are very common in this genus (Bartish et al., 2001), as further confirmed by Nybom and Bartish (2007) based on RAPD analysis. The number of *Cotoneaster* species described is progressively increasing (80 species, Rehder, 1927; 176 species, Flinck and Hylmö, 1966; 261, Phipps et al., 1990), and the latest monograph by Fryer and Hylmö (2009) has added c. 70 “new species,” bringing the total number known to approximately 400.

Previous studies have proposed that many hybridization events may have occurred in *Cotoneaster* (Fryer and Hylmö, 2009; Dickoré and Kasperek, 2010). Nonetheless, no sufficient genetic evidence has been provided for the natural hybridization occurring in this genus. Based on the phylogenetic tree constructed from three combined chloroplast regions (Li et al., 2014), 56 *Cotoneaster* species were divided into two main clades: one clade consisting of most species with erect red or pink petals, while the other clade comprised species with spreading white petals. However, it is apparent that many *Cotoneaster* species in that report exhibit intermediate morphological characters, and there is discordance between chloroplast and nrITS trees for 14 species. Nevertheless, it is difficult to identify parental species based on phylogenetic trees. First, it is difficult to collect all *Cotoneaster* species, which in many cases are morphologically undistinguished in the field. In the study by Li et al. (2014), only a small portion of the hundreds of described species in China were collected. Second, radical evolution, polyploidy and apomixis also create comb structures in the phylogenetic trees, making it even more difficult to identify their parental species.

**Figure 1:**
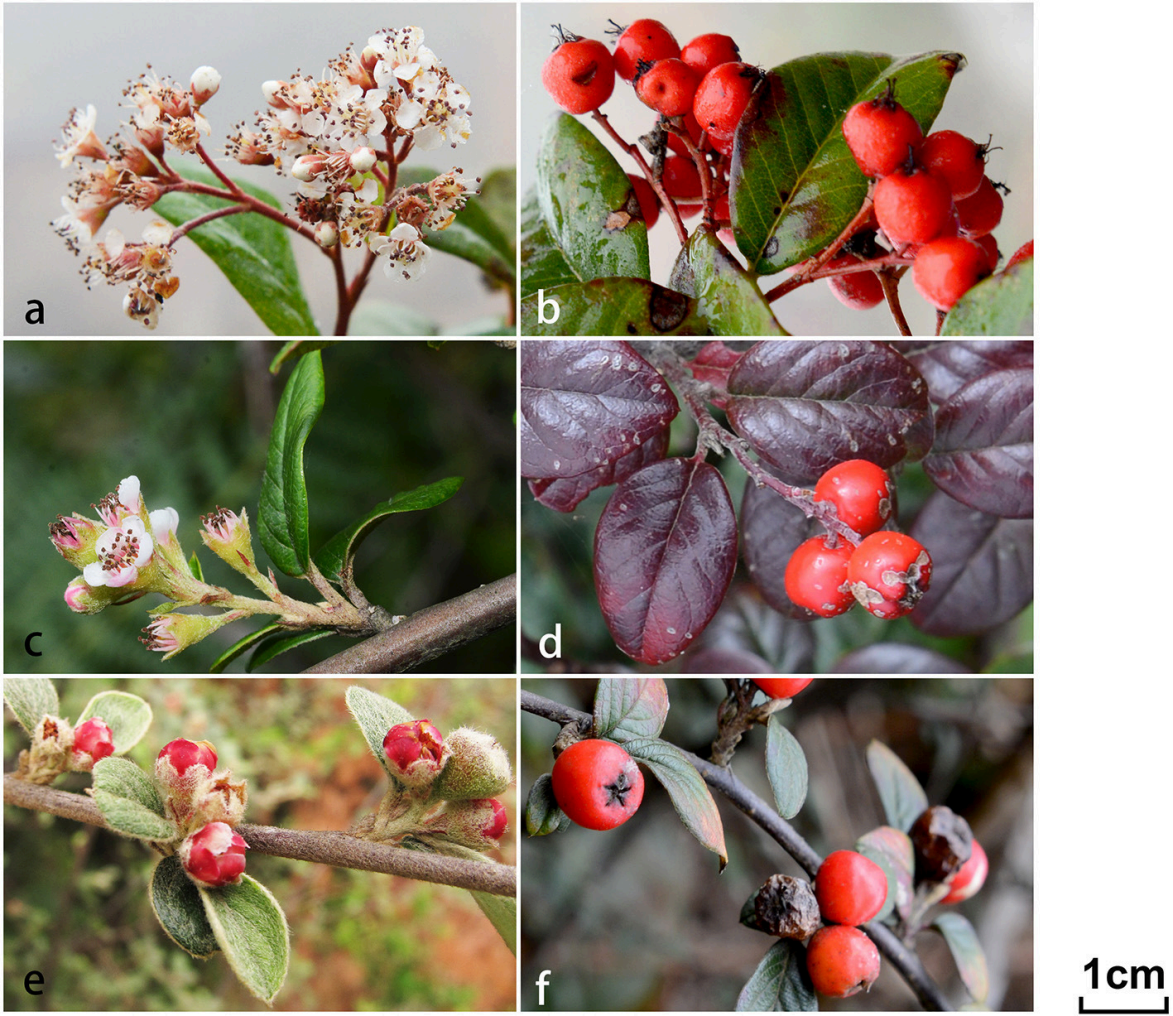
**Morphological illustrations for three ***Cotoneaster*** taxa, ***C. dielsianus*** (a,b)**, putative hybrid **(c,d)**, and *C. glaucophyllus* **(e,f)** investigated in this study. Flowers and fruits of three taxa are shown in the six frames.

In this study, we focused our objectives on a limited area (approximately 50 km^2^) of Malipo county, Yunnan, China, where a species with erect red petals (identified as *C. dielsianus*), a species with erect pink petals (*C. franchetii*), a species with spreading white petals (*C. glaucophyllus*), and an unidentified taxon with intermediate characteristics between *C. dielsianus* and *C. glaucophyllus* (the putative hybrid) can be found (Figure 1; Table 1). To validate the hybridization between *C. dielsianus* and *C. glaucophyllus*, we collected population samples for the three *Cotoneaster* species and the putative hybrid. Flow cytometry was applied to estimate their ploidy level, and then five low-copy nuclear genes and six chloroplast DNA fragments were sequenced for all samples. Through these efforts, we endeavored to answer the following questions: (1) Are these *Cotoneaster* species diploid or polyploid? (2) Are the morphologically intermediate individuals really hybrids o *C. dielsianus* and *C. glaucophyllus*? (3) If so, is the hybridization unidirectional? What is the make-up of the hybrid zone with respect to classes (i.e., F1, backcross and complex hybrid derivatives)? (4) Did the species *C. franchetii* participate in the hybridization? Based on the results, we further discussed factors contributing to the hybridization events, consequences and possible mechanism of the formation of hybrids between the parent species.

**Table 1:** **Comparison of morphological characteristics among putative hybrid, ***C. dielsianus*** and ***C. glaucophyllus*****.

**Morphological characters**	***C. dielsianus***	**Putative hybrid**	***C. glaucophyllus***
Habit	Deciduous shrub, 1–2 m	Semi-evergreen shrub, 1–2 m	Semi-evergreen shrub, 2–5 m
Lower surface of leaf	Tomentose	Pubescent	Pubescent when young, soon glabrescent
Number of pyrenes	3–5	2	2
Flowers per cyme	3–7	4–10	10–50
Petal characters	Erect	Semi-spreading	Spreading
Petal color	Red	Pinkish white	White
Ploidy level	4	4	2
2C DNA (pg, mean ± SD)	2.05 ± 0.126	2.02 ± 0.023	1.09 ± 0.034

## Methods

### Sampling

Based on the principal morphological characteristics of leaf blade size, number of flowers per cyme, petal characters and petal color (Figure 1; Table 1; Fryer and Hylmö, 2009), at least 18 individuals were collected for each of the three *Cotoneaster* taxa and the putative hybrid from Malipo county, Yunnan, China (Table 2). In addition, one congeneric species, *C. frigidus*, was sampled in Tibet and used as a outgroup (Table 1). For each individual, fresh leaves were collected and deposited in silica gel in zip-lock plastic bags for DNA extraction. Voucher specimens were stored in the Herbarium of Sun Yat-sen University (SYS).

**Table 2:** **Sampling detail of putative hybrid groups, relative and outgroup species (***C.frigidus***) used in this study**.

**Taxon**	**Collecting number (DNA sample no.)**	**Geographical origin (China)**	**Coordinates (N, E)**	**Altitude (m)**
Putative hybrid	13917 (01–30)	Malipo County, Yunnan	23.13°, 104.80°	1,900–2,159
*C. dielsianus*	13916 (31–48)	Malipo County, Yunnan	23.13°, 104.80°	2,159
*C. franchetii*	13915 (49–70)	Malipo County, Yunnan	23.13°, 104.80°	2,159
*C. glaucophyllus*	13949 (71–96)	Malipo County, Yunnan	23.18°, 104.82°	1,501–2,159
*C. frigidus*	14650	Jilong County, Tibet	28.43°, 85.26°	2,972

### DNA extraction, primer design, PCR, and sequencing

Total genomic DNA was extracted from dried leaf tissue using a modified CTAB method (Doyle and Doyle, 1987). According to Duarte et al. (2010), a total of 959 single copy nuclear genes were identified based on comparison of the genomes of *Arabidopsis thaliana, Populus trichocarpa, Vitis vinifera* and *Oryza sativa*, and the sequences for these 959 genes in *Arabidopsis* were downloaded. Of these, 640 obtained BLASTN hits in the cDNA library of *Malus domestica* (not shown) with a cut-off *e*-value of 1e^−10^, and sequences for the top hits were extracted and identified as putative single-copy genes in *M. domestica*. Of these, 33 paired PCR primers for exon-primed, intron-crossing (EPIC) amplifications were designed from randomly selected sequences using Primer Premier 6.0 (PREMIER Biosoft International, Palo Alto, CA, USA). Among these, five were widely amplified in *Cotoneaster* with a single clear band and obtained good sequencing results. Annotations using BLASTX against the NCBI non-redundant protein database showed that three (DUF, UPF, and WD) significant hits. Six chloroplast regions were selected: *ndh*F, *rpl*16, *rps*16, *trn*C-*ycf* 6, *trn*G-*trn*S, and *trn*H-*rpl*2 (Campbell et al., 2007; Lo and Donoghue, 2012). Primers and annotation for all nuclear and chloroplast genes are shown in Table 3.

**Table 3:** **Primers of five low-copy nuclear genes based on ***Malus domestica*** genome and six universal chloroplast fragments**.

**Locus**	**Primer sequences (5′–3′)**	**Length (bp)**	**ID for apple coding sequence/reference**	**Score (*E*-value)**
DUF	f:ACAAGTCCAATGCCAATGA	840	MDP0000336096	152(7e-36)
	r:AATATGCCGTAGCCTCCTA			
NA1	f:GCTGGATCACGACTGAGATAAG	568	MDP0000144617 and MDP0000246780	285(3e-76) and 127(2e-28)
	r:TTGTTGAAGCCTCATTCTCTGG			
NA2	f:CCTTTCTCCACTGGGTTAA	461	MDP0000130385	258(8e-68)
	r:GCACTTGAGGTAGCATAATAG			
UPF	f:CAGACTGCTGCCATAATAGA	645	MDP0000174677 and MDP0000940113	127(1e-28) and 222(5e-57)
	r:TAGAAGTAATCGCCACAGAG			
WD	f:GTTCCTCTATCATCACCAGTT	811	MDP0000283138	222(6e-57)
	r:ACCAGTGCCAAGTCTATTC			
*ndh*F	2f:ACTCATGCTTATTCGAAAGC	1036	Campbell et al., 2007	
	1.6r:CCTACTCCATTGGTAATTCCAT			
*rpl*16	f71:GCTATGCTTAGTGTGTGACTCGTTG	891	Campbell et al., 2007	
	r1516:CCCTTCATTCTTCCTCTATGTTG			
*rps*16	f:GTGGTAGAAAGCAACGTGCGACTT	662	Campbell et al., 2007	
	r2:TCGGGATCGAACATCAATTGCAAC			
*trn*C-*ycf*6	f:GCTTGATTCTAAGTATCTGGG	648	Design base on NCBI data (Lo and Donoghue, 2012)	
	r:CAACACCGTTGATGAAACA			
*trn*G-*trn*S	f:CGTGTTGTATCAGAGAACC	415	Identical with *trn*C-*ycf*6	
	r:TTTCATCCGAGAGTGCTTT			
*trn*H-*rpl*2	f:TCTTCGTCGCCGTAGTAA	316	Identical with *trn*C-*ycf*6	
	r:AAGGCAGTGGATTGTGAAT			

PCR reactions were conducted in 20 μL total volumes containing 25 ng of template DNA, 2 μL of 10 × Mg^+2^FreeBuffer, 1.0 mM MgCl2, 0.2 mM each dNTP, 0.2 μM each primer, and 1 unit Taq DNA polymerase (Apex Bioresearch Products, Research Triangle Park, NC, USA). The amplifications were performed using the following conditions: initial denaturation at 94°C for 4 min, followed by 35 cycles of 94°C for 30 s, an annealing temperature of 55°C for 30 s, 72°C for 1 min, and a final extension of 72°C for 10 min. The PCR products were purified by electrophoresis on a 1.2% agarose gel, followed by extraction using a Pearl Gel Extraction Kit (Pearl Biotech, Guangzhou, China). The purified PCR products were then sequenced on an ABI 3730 DNA Analyzer with the BigDye Terminator Cycle Sequencing Ready Reaction Kit (Applied Biosystems, Foster City, CA). All sequences were deposited in GenBank with the accession numbers KY469293-KY470828.

### Ploidy determination

For the 96 individuals sampled, the ploidy of putative hybrid, *C. dielsianus* and *C. glaucophyllus*, were obtained using four randomly selected individuals in each taxon. The ploidy of silica-dried leaf material was determined by flow cytometry analysis (performed by the Flow Cytometry Lab in Benaroya Research Institute at Virginia Mason, USA) using a modified version of the hand-chopping method described by Roberts et al. (2009). For each sample, approximately 4 mg of dried leaf material and one drop of chicken erythrocyte nuclei (2.5 pg/2C) as the internal standard were finely chopped using a single-edged razor blade in 1000 μL of cold lysis buffer [0.1 M citric acid, 0.5% v/v Triton X-100, 1% w/v PVP-40 (polyvinylpyrrolidone, average molecular weight 40,000)] (Yokoya et al., 2000; Hanson et al., 2005) in a petri dish on a cold chopping surface. After 5 min of incubation on ice and intermittent gentle mixing by pipetting up and down, each sample was filtered using a 5-mL polystyrene round-bottomed tube with a cell-strainer cap (BD Falcon; Becton Dickinson and Co., Franklin Lakes, NJ, USA). A 140-μL aliquot of filtrate was placed in a new 1.5-mL Eppendorf tube with 1 μL of RNaseA (1 mg mL^−1^) (Thermo Scientific Molecular Biology, Fisher Scientific, Pittsburgh, PA, USA) and incubated at room temperature for 30 min. Next, 350 μL of propidium iodide (PI) staining solution (0.4 M NaPO_4_, 10 mM sodium citrate, 25 mM sodium sulfate, 50 μg mL^−1^ PI) was added to each tube of nuclei suspension. After 1 h at room temperature, the stained nuclei suspensions were analyzed at 14 μL min^−1^ on an Accuri C6 flow cytometer (BD Biosciences, San Jose, CA, USA) fitted with a 488-nm laser. Fluorescence measurements were made using the FL2 (585/40 nm) optical filter, capturing 10 000 events and utilizing the FL2-A values for the 2C peak.

### Sequence analysis

The obtained sequences were edited and analyzed by Geneious R8 software (Biomatters, Ltd., Auckland, New Zealand). To determine possible copy numbers in the genome, a BLASTN search against apple genome databases (http://www.rosaceae.org/species/malus/malus_x_domestica/genome_v1.0) was performed with a bit score threshold of >100 and a cut-off *E*-value of 1e^−6^. No more than two hits were detected in each of the five investigated genes (Table 3), indicating that they were single-copy or low-copy regions in the genome. Furthermore, an additional 2–3 pairs of primers were developed to anchor different sites for each nuclear genes (data not shown). We obtained identical sequences using fragments from PCR products traced by these primers, confirming that they are very likely orthologous in *Cotoneaster*.

Polymorphisms at variable sites were identified as superimposed nucleotides (additive patterns) from chromatograms of direct sequences (Whittall et al., 2000), and indel polymorphisms were determined by reading the sequence chromatogram in both directions. At the five nuclear genes, we phased the haplotypes using DnaSPv5 (Librado and Rozas, 2009), and used Network 5001 (www.fluxus-engineering.com) to resolve the relationships of the haplotypes with the median-joining method (Bandelt et al., 1999).

For each nuclear gene and combined chloroplast datasets, we reconstructed the phylogeny of the haplotypes using maximum parsimony (MP) and maximum likelihood (ML) methods, as estimated by PAUP4.0b (Swofford, 2001). For parsimony analyses, a heuristic search with tree bisection-reconnection branch swapping, the MulTrees option, accelerated transformation optimization, and 100 random addition replicates was implemented. We defined indels as the fifth state and each indel with two or more nucleotides as a single mutational event. One thousand bootstrap replicates were computed with maxtrees being set to 500. For ML analysis, we selected an appropriate nucleotide substitution model for each gene based on the result of Modeltest 3.7 (Posada and Buckley, 2004). Best-fit models based on the Hierarchical Likelihood Ratio tests (hLRTs) in Modeltest were calculated (Table S1); four of the six models were F81 and the other was HKY+G. Similarly, ML analysis was performed using a heuristic search with tree bisection-reconnection branch swapping, holding one tree at each step. Node support was estimated with 1000 bootstrap replicates and the maxtrees was also set to 500.

## Results

The aligned sequences of the five nuclear genes and six chloroplast regions obtained from all individuals of *C. dielsianus, C. glaucophyllus, C. franchetii* and the putative hybrid are shown in Table 3. The shortest aligned length among the 11 makers is 316 bp (*trn*H-*rpl*2), while the longest is 1036 bp (*ndh*F). Considerable sequence variations and high divergence were detected in these four taxa among the 11 fragments (Table 4). Surprisingly, all individuals of the putative hybrid shared identical sequences.

Table 4**Fixed nucleotide substitutions and gaps between putative hybrid, ***C. dielsianus, C. glaucophyllus***, and ***C. franchetii*** in the five nuclear genes (DUF, NA1, NA2, UPF, and WD) and six chloroplast genes (***ndh***F, ***rpl***16, ***rps***16, ***trn***C-***ycf***6, ***trn***G-***trn***S, ***trn***H-***rpl***2)**.

**Table:** 

**Gene**	**DUF**														**NA1**			
Site (bp)	66	117	266	285	412	454	532	571	581	607	649	672	766	810	379	397	435	443
Putative hybrid	S	W	Y	W	Y	Y	R	Y	S	Y	R	W	Y	K	Y	K	W	K
*C. dielsianus*	G	A	T	A	C	T	A	C	G	C	A	A	C	G	C	T	A	G
*C. glaucophyllus*	C	T	C	T	T	C	G	T	C	T	G	T	T	T	T	G	T	T
*C. franchetii*	S	A	Y	W	Y	Y	R	Y	S	Y	R	T	C	K	Y	K	W	K
						**NA2**					**UPF**						**WD**	
517	520	521	525	526	527	149	215	268	340	408	187	195	257	278	623	625	40-45	364
R	R	R	W	W	R	R	Y	K	S	R	Y	Y	R	W	K	K	——/ TCACAT	M
G	A	A	T	A	G	G	C	T	C	G	C	C	A	T	G	G	——	A
A	G	G	A	T	A	A	T	G	G	A	T	T	G	A	T	T	TCACAT	C
R	R	R	W	W	R	R	Y	K	S	R	C	C	A	T	K	K	——/ TCACAT	M

**Table:** 

**Gene**	***ndh*****F**		***rpl*****16**			***rps*****16**			***trn*****C-*****ycf*****6**		***trn*****G-*****trn*****S**				***trn*****H-*****rpl*****2**		
Site (bp)	379	716	582	589	635	67	90	651	259	373	44	109	188-190	191-219	19	59-92	254
Putative hybrid	A	C	G	T	G	T	T	G	A	C	T	G	TTC	TTTATTCCTTTTATTTTAGTTAAAGTAAA	A	ATAAATATTTAATATAAATATTAAATATAAATGG	A
*C. dielsianus*	T	A	T	G	A	C	C	T	G	G	−	T	CCT	−−−−−−−−−−−−−−−−−−	C	TTAAA −−−−−−−−−−−−−	A
*C. glaucophyllus*	A	C	G	T	G	T	T	G	A	C	T	G	TTC	TTTATTCCTTTTATTTTAGTTAAAGTAAA	A	ATAAATATTTAATATAAATATTAAATATAAATGG	A
*C. franchetii*	A	C	T	G	A	T	T	G	A	C	−	G	TTC	TTTATTCCTTTTATTTTAGTTAAAGTAAA	A	−−−−−−−−−− AATATAAATATTAAATATAAATGG	C

### Sequence analyses of the five nuclear genes

The aligned lengths and number of variable sites at the five nuclear genes in the four taxa are shown in Tables 3, 4, respectively. There were a total of 36 fixed nucleotide substitutions and one fixed 6-bp indel (insertion/deletion) between *C. dielsianus* and *C. glaucophyllus* across the whole nuclear gene data set. All individuals of the putative hybrid showed chromatogram additivity at these fixed sites. *C. franchetii* also showed chromatogram additivity at 29 fixed variations and one fixed 6-bp indel between *C. dielsianus* and *C. glaucophyllus*.

**Figure 2:**
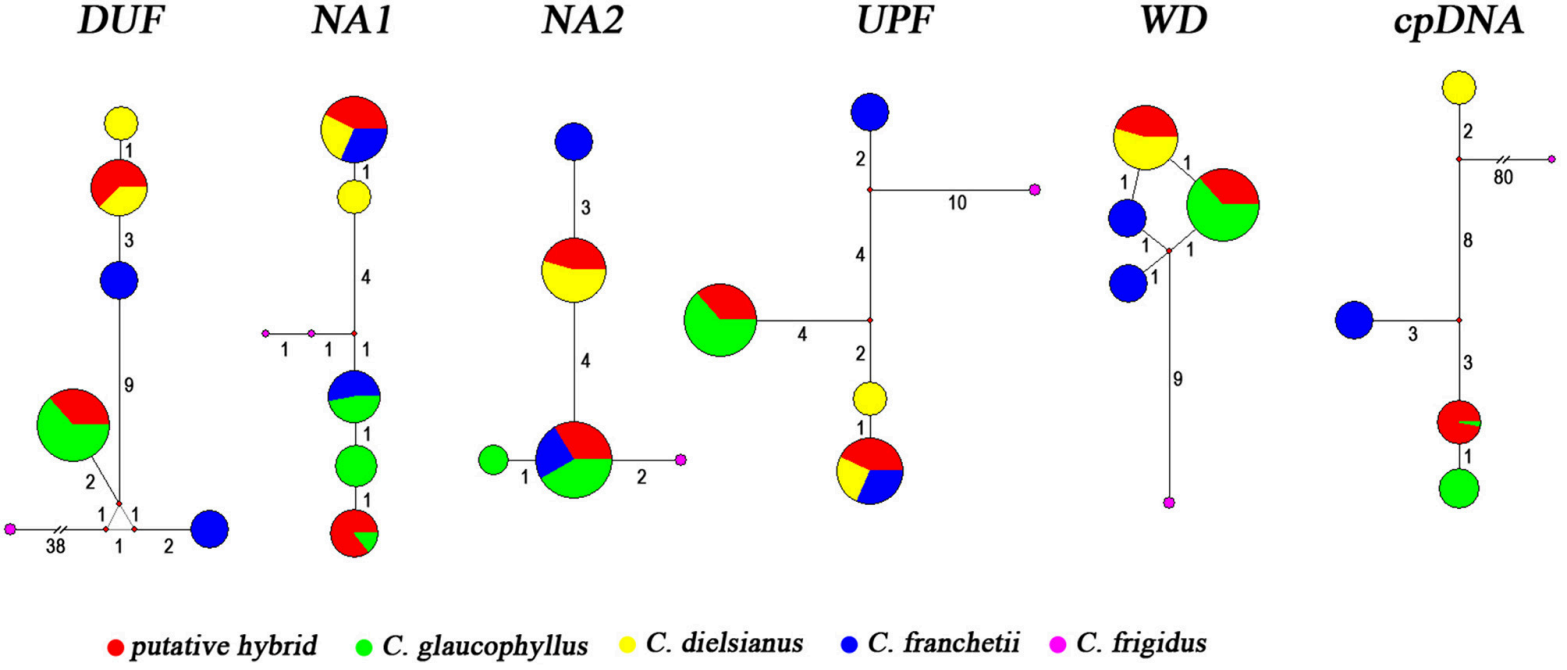
**Haplotype networks of five nuclear genes and six cpDNAs for putative hybrid, ***C. dielsianus***, ***C. glaucophyllus***, and ***C. franchetii*****. Mutation steps are shown by the length of the connecting lines.

In the haplotype analysis, each taxon exhibited a low level of haplotype diversity, and no more than three haplotypes were observed for each of the five genes (Figure 2, Table 4). *C. dielsianus* possessed 1–2 haplotypes at each gene: 1 haplotype observed at NA2 and WD and 2 haplotypes each at DUF, NA1 and UPF. *C. glaucophyllus* harbored 1–3 haplotypes at each gene: 3 haplotypes observed at NA1; 2 haplotypes at NA2; and 1 haplotype each at DUF, UPF and WD. Nevertheless, no haplotype was shared between the two species. Each individual of the putative hybrid had two haplotypes for each gene: one was shared with *C. dielsianus* and the other was shared with *C. glaucophyllys*. For the other species, *C. franchetii*, one haplotype at NA1 and NA2 was shared with *C. glaucophyllus*, the other haplotype at NA1 and UPF was shared with *C. dielsianus*, and the other six were unique.

**Figure 3:**
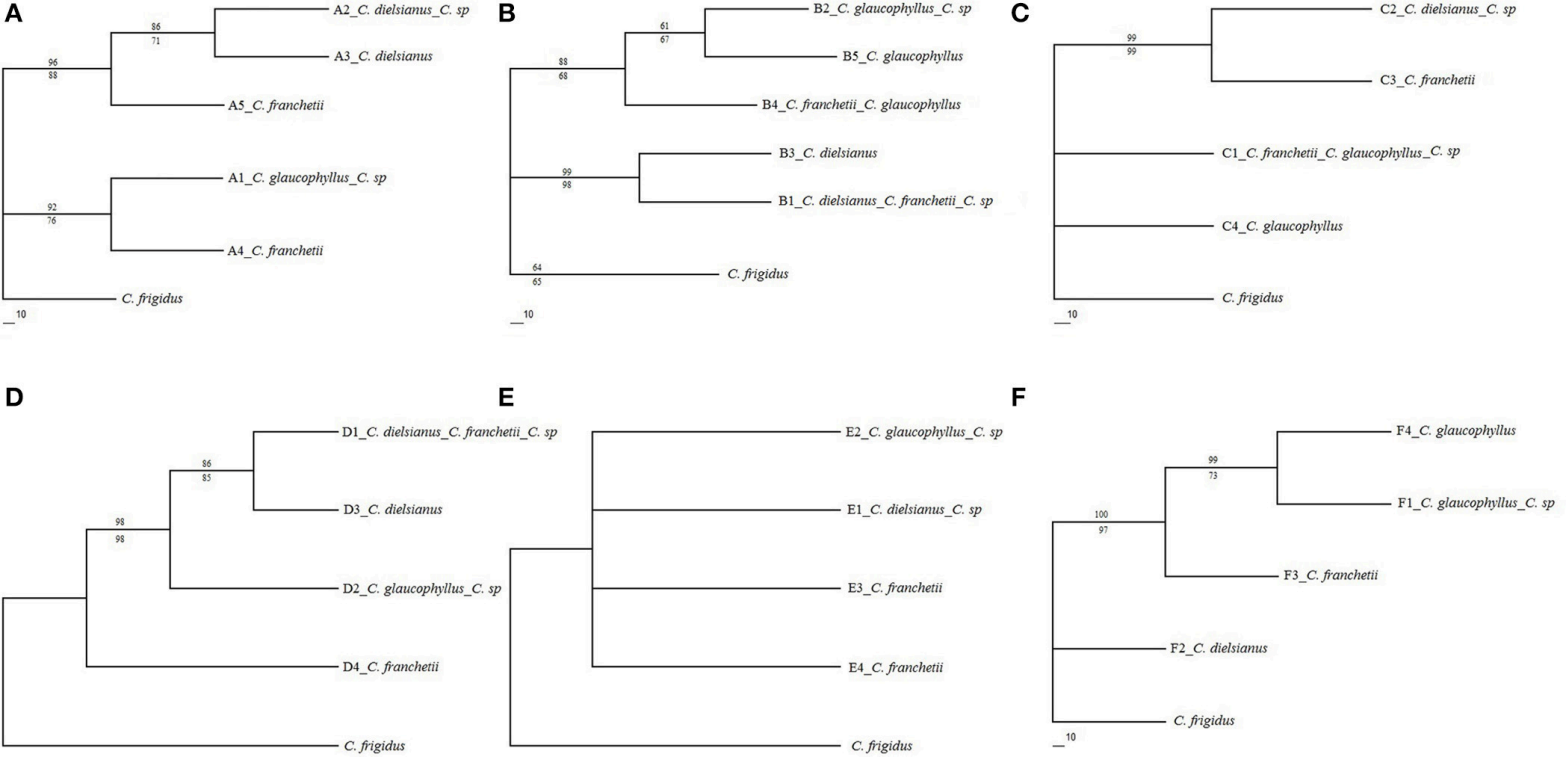
**Phylogenetic analyses of haplotype data for five nuclear genes (A–E)** and six cpDNAs for the four taxa of *Cotoneaster* (**F**; *C*. sp represents putative hybrid), rooted with outgroup *C. frigidus*. Numbers above and below branches indicate maximum parsimony and maximum likelihood bootstrap values (>50%).

MP and ML algorithms were used to construct phylogenetic trees (Figure 3) using the haplotypes at each nuclear gene. The number of parsimony-informative characteristics, steps and values of CI, RI, and RC with the MP algorithm were shown in Table S1 (see Supplementary Material). In the MP tree (bootstrap values above branches), the two haplotypes of the putative hybrid, identical to *C. dielsianus* and *C. glaucophyllus*, formed two well-separated clades. The two haplotypes of *C. franchetii* were also well-separated clades and tended to gather the two haplotypes of putative hybrid. The topologies of these trees were the same as those generated using the ML analysis (bootstrap values below branches).

### Sequence analyses for the combined chloroplast regions

The aligned length of the six concatenated chloroplast fragments in *C. dielsianus, C. glaucophyllus, C. franchetii* and the putative hybrid was 3,968 bp (Table 3). A total of 14 fixed nucleotide substitutions and three fixed indels were detected between *C. dielsianus* and *C. glaucophyllus*. No within-species polymorphism was detected in the putative hybrid, *C. dielsianus* or *C. franchetii* (Figure 3). *C. glaucophyllus* had two closely related haplotypes and in the tree, these two haplotypes were gathered together and well separated from *C. dielsianus* (Figure 3). All sequences of the putative hybrid were identical to those of *C. glaucophyllus*. For *C. franchetii*, the single haplotype was unique.

### Ploidy of *C. dielsianus, C. glaucophyllus*, and their putative hybrid

The ploidy of the putative hybrid, *C. dielsianus* and *C. glaucophyllus*, was obtained using four randomly selected individuals in each taxon. The average 2C-values/genome sizes are shown in Table 1. As Kroon (1975) reported, the examined seed stocks and chromosome numbers of 28 species in *Cotoneaster* were determined, including *C. dielsianus*, which has a chromosome count of 2*n* = 68 (tetraploid). In addition, based on a previous study by Folta and Gardiner (2009), 2C-values and ploidies for *C. melanocarpa* were provided (2C-value = 2.24, tetraploid); these values are close to the 2C-values of *C. dielsianus* and the putative hybrid in our results (2.05 ± 0.126; 2.02 ± 0.023). The consistency of these data indicate that our results are credible and it was inferred that the putative hybrid and *C. dielsianus* were tetraploid. As the 2C-value of *C. glaucophyllus* was estimated to be 1.09 ± 0.034, it was inferred that *C. glaucophyllus* was diploid.

## Discussion

### Molecular identification of natural hybridization between *C. dielsianus* and *C. glaucophyllus*

The application of low-copy nuclear genes in combination with chloroplast regions has become an efficient way to validate hybridization events. Many hybrids have been proposed and validated, including *Melastoma* (Dai et al., 2012), *Acrostichum* (Zhang et al., 2013), *Eriobotrya* (Fan et al., 2014), and *Ilex* (Shi et al., 2016). In this study, we collected multiple individuals for each *Cotoneaster* species observed in the small confined area (southeastern Malipo, approximately 50 km^2^). Five low-copy nuclear genes and six chloroplast regions were sequenced to validate the hybridization between *C. diesianus* and *C. glaucophyllus*.

Our molecular data support the hypothesis that *C. dielsianus* and *C. glaucophyllus* are two distantly related species, between which a total of 50 fixed nucleotide substitutions and four fixed indels were identified across 11 investigated genes. The putative hybrid was identified as chromatogram additivity between *C. dielsianus* and *C. glaucophyllus*, as observed for all the individuals of the putative hybrid. All hybrid individuals likely arose from an initial F1 individual and its derivatives via apomixis, since each individual harbored two haplotypes at each of the five nuclear genes that were matched with *C. dielsianus* and *C. glaucophyllus*. Further, based on the chloroplast sequence data, *C. glaucophyllus* obviously served as the maternal species for all investigated individuals of the hybrid.

*C. franchetii* did not participate in the formation of the hybrid, as most of its haplotypes at the nuclear level are unique (Table 4). Surprisingly, *C. franchetii* showed chromatogram additivity in many differentially fixed sites between *C. glaucophyllus* and *C. dielsianus*. Furthermore, it always possessed two haplotypes for each nuclear gene, and the genotypes of all five nuclear genes from all individuals were identical to each other. This evidence indicated that *C. franchetii* might also be a hybrid taxon and that all the individuals were F1s, yet neither *C. dielsianus* nor *C. glaucophyllus* could serve as its parent species, as *C. franchetii* harbored many unique haplotypes and one variable site that was not shared by *C. dielsianus* or *C. glaucophyllus*. However, its hybrid status and parentage are beyond the scope of this study. Further studies are needed to confirm its hybrid origin and parentage.

It is interesting that all the investigated individuals of the hybrid are F1s, with the same genotypes for all the nuclear genes, and that the hybrid samples detected are tetraploid. It is possible that natural hybridization between *C. glaucophyllus* and *C. dielsianus* could have produced many F1s, but that most disappeared quickly and only one tetraploid F1 individuals was successful and it produced many progeny through apomixis. This phenomenon would be in accord with previous studies showing that many apomictic taxa are of allopolyploid origin (Robertson et al., 2010; Sochor et al., 2015). *C. dielsianus* was mostly tetraploid, while *C. glaucophyllus* was mostly diploid, and thus the formation of the tetraploid hybrid may arise from the cross of a 2n gamete produced by through apomeiosis of *C. glaucophyllus* and the other 2n gamete produced from normal meiosis of *C. dielsianus*. This explains why the hybridization was unidirectional, as all of the hybrid individuals are apomictic progeny of a single individual, in which *C. glaucophyllus* serves as maternal species.

In addition, the combined chloroplast haplotype of the hybrid individuals was identical to the minor haplotype of *C. glaucophyllus* (H_A_; only one individual has this haplotype). One explanation for this phenomenon is that hybridization between the two species is a very rare event. Another explanation is that F1 hybrids containing the H_A_ chloroplast haplotype may have some advantages for survival in the disturbed environment.

### Factors contributing to natural hybridization between *C. dielsianus* and *C. glaucophyllus*

**Figure 4:**
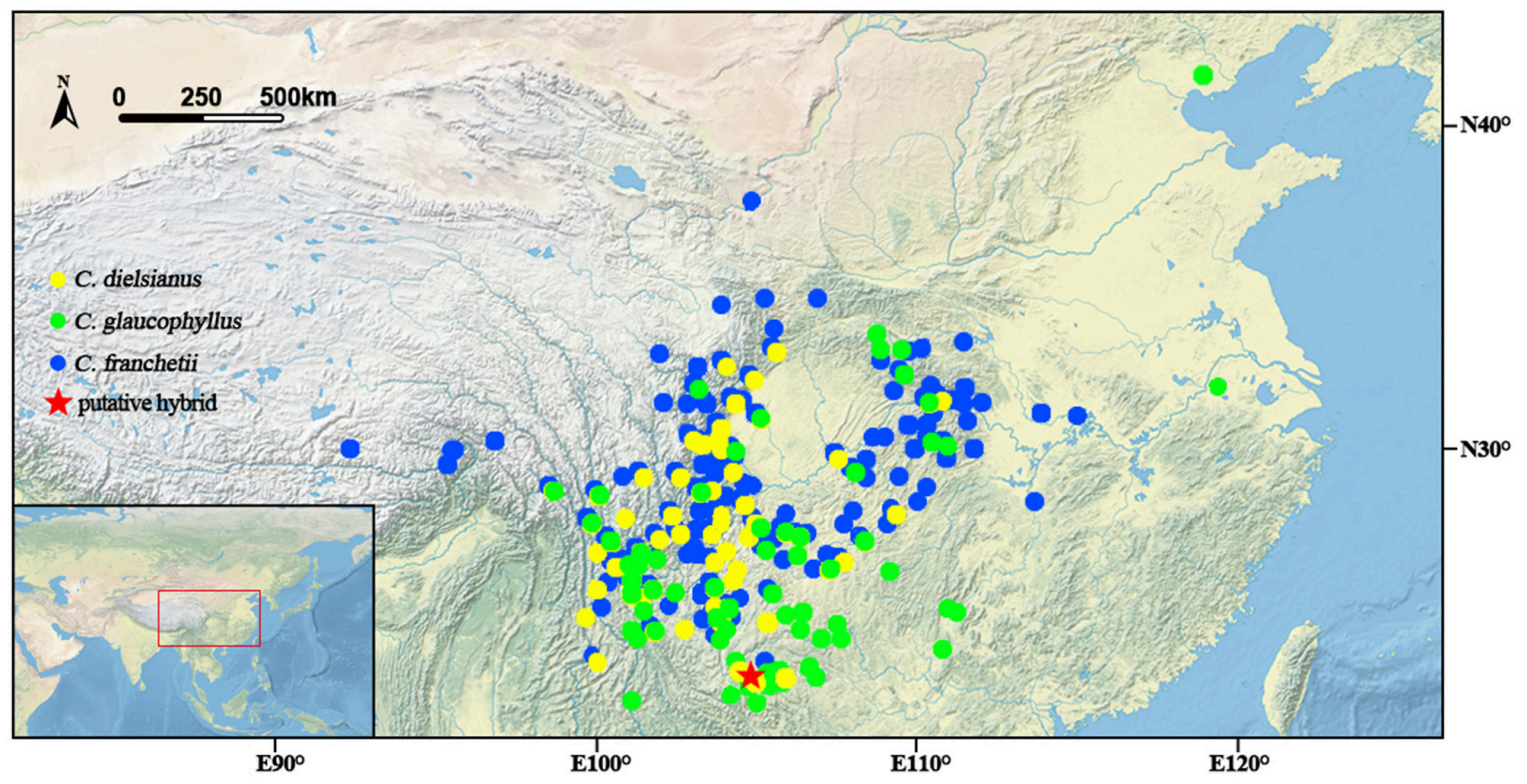
**Distribution of three taxa of ***Cotoneaster*** investigated around China according to the Chinese Virtual Herbarium database (CVH^**1**^) and location of the putative hybrid**.

*C. dielsianus* and *C. glaucophyllus* overlap significantly in geographic distribution (Figure 4) and share the same flowering periods from June to July, despite their distinct flower morphologies. The two species differ in petal color and shape, and may attract different pollinators: *C. dielsianus* displays red and erect petals which are more attractive to butterflies and moths, whereas *C. glaucophyllus* presents white, spreading petals that are preferable to bees and flies (Lovell, 1902). These phenomena were in agreement with our field observations. Furthermore, the two species prefer different habitats: *C. dielsianus* mainly occurs in sparse forests, while *C. glaucophyllus* is always found on cliffs and steep slope (personal observations; Lu and Brach, 2003).

In the study area of southeastern Malipo, which is close to the border with Vietnam, intensive agricultural development began in the 1950s and has resulted in widespread fragmentation of forests due to severe logging (Tang et al., 2011). The hybrid and three other *Cotoneaster* species were found in a deserted grass land where the primitive forest was destroyed approximately 50 years ago. However, in another location (Weixi county, Yunnan, China; 27.56° N, 99.03° E), both *C. dielsianus* and *C. glaucophyllus* were found on a rather steep slope with sparse forests. No hybrids were found. It appears that anthropogenic disturbances may be the major factor promoting hybridization between *C. dielsianus* and *C. glaucophyllus*, leading to mixing of previously distinct gene pools (Arnold, 1997). This provides an opportunity for the two species to contact each other. More importantly, it can create a new ecological niche in which the hybrid can establish its populations.

### Consequences of hybridization between *C. dielsianus* and *C. glaucophyllus*

In this study, all sampled hybrids could be produced through hybridization and subsequent apomixis, and no sign of backcross and introgression was detected between the hybrid and its parental species, based on the 11 investigated genes. It is very likely that this tetraploid hybrid may not be able to produce viable progeny through sexual reproduction in most cases. This also matched the common observation that genome doubling reduces or eliminates the possibility of new polyploid backcrossing with its parents (Soltis and Soltis, 2009). Therefore, genetic isolation can maintain the species integrity of *C. dielsianus* and *C. glaucophyllus* despite hybridization.

The distribution range of the new-born hybrid *C. dielsianus* × *C. glaucophyllus* is currently very limited. However, the distribution for allopolyploidy could also be very widespread. For example, for *C. franchetii*, we inferred another allopolyploidy whose individuals are widely distributed in China and other countries. Previous studies also showed that many invasive species exhibited allopolyploidy, such as *Spartina anglica, Viola riviniana* and *Rhododendron ponticum* (Ellstrand and Schierenbeck, 2000). Attentions should be focused on the future development of *C. dielsianus* × *C. glaucophyllus*, which may eventually threaten other *Cotonearster* species.

### Conservation implications

Hybridization, in combination with polyploidy and apomixes, has produced numerous novel phenotypes, leading to increasing numbers of species in Rosaceae. A key element for understanding these agamic complexes is the identification of diploid sexual taxa. These are the foundations of the complex (Bayer and Stebbins, 1987), and it is very important to pay attention to the diploid species whose current species status could be threatened by the increasing polyploidy number. However, the ploidy level of many species is still unknown in most genera of Rosaceae (Dickinson et al., 2007). In this study, dried leaves were used to estimate the ploidy level of these *Cotoneaster* species via flow cytometry, in which a large volume of cold lysis buffer was added to reduce the viscosity of the chopped tissue suspension before filtering (Talent and Dickinson, 2005). By comparison with the conventional method of chromoson counting, this technology appears to be more convenient and economical, especially when fresh tissue is not available, and will serve as a reference for the evaluation of the ploidy of other Roseaceae species.

## Summary

In this study, the sequence and haplotype analyses of five low-copy nuclear genes and six chloroplast regions, in combination with ploidy level analysis, provided convincing evidence for the hybridization of *C. dielsianus* (tetraploid) and *C. glaucophyllus* (diploid), in which all *C. dielsianus* × *C. glaucophyllus* individuals (allotetraploid) were identified as F1s and harbored identical sequences, indicating that they were produced via apomixis by a single F1 individual. We have shown that hybridization, polyploidy and apomixis lead to astonishing complexities in Roseaceae. Our study also provides a reliable way to screen and validate hybridization events occurring in this family. The five low-copy nuclear genes and the screening method may also be useful in these studies.

## Author contributions

ML analyzed data and wrote the paper, QF and SC designed the research, and RZ, FL, and WL revised the paper. All authors approved this manuscript for publication.

### Conflict of interest statement

The authors declare that the research was conducted in the absence of any commercial or financial relationships that could be construed as a potential conflict of interest.

## Footnotes

^1^CVH (2014). *Chinese Virtual Herbarium*. Available online: http://www.cvh.ac.cn/ (accessed on 10 May 2014).
