# Supplementary material for: Molecular Evidence for Natural Hybridization between Cotoneaster dielsianus and C. glaucophyllus
Source: Front Plant Sci. 2017 May 9;8:704. doi: 10.3389/fpls.2017.00704 (PMC5422516; doi:10.3389/fpls.2017.00704)
Supplement: Supplementary file 1 [file Table1.docx]

**SUPPLEMENTARY MATERIAL**

**Table S1 | Best-fit model based on the hierarchical likelihod ratio tests (hLRTs) for ML analysis, number of parsimony-informative characteristics (PICs), steps and values of CI, RI and RC with the MP algorithm, and haplotypes of four *Cotoneaster* taxa for each nuclear gene and six cpDNA*.* The number in parentheses following the haplotype indicates the number of individuals with this haplotype in the gene.**

|  |  |  | **Marker** |  |  |  |
| --- | --- | --- | --- | --- | --- | --- |
|  | **DUF(A)** | **NA1(B)** | **NA2(C)** | **UPF(D)** | **WD(E)** | **cpDNA(F)** |
| Model | HKY+G | F81 | HKY | F81 | F81 | F81 |
| PICs | 13 | 9 | 5 | 6 | 2 | 56 |
| steps | 60 | 12 | 13 | 26 | 87 | 188 |
| CI\RI\RC | 0.950\0.850\0.808 | 1.000\1.000\1.000 | 1.000\1.000\1.000 | 1.000\1.000\1.000 | 0.977\0\0 | 0.957\0.857\0.821 |
| *C. sp* | A1(30);A2(30) | B1(30);B2(30) | C1(30);C2(30) | D1(30);D2(30) | E1(30);E2(30) | F1(31) |
| *C. dielsianus* | A2(18);A3(18) | B1(18);B3(18) | C2(36) | D1(18);D3(18) | E1(36) | F2(18) |
| *C. glaucophyllus* | A1(52); | B2(5);B4(19);B5(28) | C1(37);C4(15) | D2(52); | E2(52) | F4(25) |
| *C. franchetii* | A4(22);A5(22) | B1(22);B4(22) | C1(22);C3(22) | D1(22);D4(22) | E3(22);E4(22) | F3(22) |
